# The efficacy of Chinese herbal medicine Buyang Huanwu Decoction combined with acupuncture to treat sequela of apoplexy: A protocol for systematic review and meta-analysis

**DOI:** 10.1097/MD.0000000000027679

**Published:** 2021-11-05

**Authors:** Jiayu Zhang, Zhaowei Li, Liang Zhang

**Affiliations:** a Medical College of Acu-Moxi and Rehabilitation, Guangzhou University of Chinese Medicine, Guangdong, China; b College Clinic, Guangzhou Health Science College, Guangdong, China; c College of Physical Education and Health, Guangzhou University of Chinese Medicine, Guangdong, China.

**Keywords:** acupuncture, Buyang Huanwu Decoction, meta-analysis, sequela, stroke

## Abstract

**Background:**

Stroke is a common disease in neurology, patients often have different degrees of sequelae, which affect the patient's quality of life. We conducted a protocol for systematic review and meta-analysis to assess the efficacy of Buyang Huanwu Decoction combined with acupuncture for the treatment of stroke sequelae.

**Methods:**

The current protocol is prepared in accordance with the Preferred Reporting Items for Systematic reviews and Meta-Analyses for Protocols statement guideline. Seven electronic databases including Web of Science, Embase, PubMed, Wanfang Data, Scopus, Science Direct, and Cochrane Library were searched in August 2021 by 2 independent reviewers. The risk of bias assessment of the included studies was performed by 2 authors independently using the tool recommended in the Cochrane Handbook for Systematic Reviews of Interventions (version 5.1.0). We will perform meta-analysis using STATA 11.0 (http://www.stata.com; Stata Corporation, College Station, TX).

**Results:**

The review will add to the existing literature by showing compelling evidence and improved guidance in clinic settings.

**Conclusion:**

Buyang Huanwu Decoction combined with acupuncture seems to be an effective therapy to ameliorate the clinical symptoms of stroke sequelae. In order to further determine the effectiveness and safety of Buyang Huanwu Decoction combined with acupuncture in treating stroke sequelae, more multicenter and prospective randomized controlled trials must be carried out.

## 1 Introduction

Stroke is a common disease in neurology, patients often have different degrees of sequelae, which affect the patient's quality of life.^[1–3]^ Stroke is currently the leading cause of death and disability in our population, and about 2 million strokes occur every year in China.^[4]^ The poststroke sequelae mainly refer to a condition left after hemorrhage of acute cerebrovascular disease, hemiplegia, numbness, skewed eyes, and poor speech.^[5,6]^ At present, stroke, cancer, and coronary heart disease have become the 3 major diseases of human death in the world, which poses a serious threat to the safety of patients’ lives and physical and mental health. In recent years, more and more reports have shown that acupuncture combined with traditional Chinese medicine has a significant effect in the treatment of sequelae of stroke, which can improve the patient's nerve function and limb function, relieve the symptoms of sequelae, and improve the quality of life of patients.^[7,8]^

In China, traditional Chinese medicine and acupuncture are commonly used to treat sequelae of stroke. Buyang Huanwu Decoction is a common prescription which can treat sequelae of stroke.^[9]^ It consists of astragalus, angelica tail, red peony root, earthworm, Chuanxiong, safflower, and peach kernel. Its traditional function is to replenish qi, activate blood circulation, and remove blood stasis.^[9,10]^

Through the analysis of the names of diseases treated by Buyang Huanwu Decoction and the bibliometrics research of modern literature, it is found that the most frequent Traditional Chinese Medicine diseases are apoplectic sequelae. At the same time, acupuncture combined with Buyang Huanwu Decoction is also common in clinical treatment of sequelae of stroke. In this study, we conducted a protocol for systematic review and meta-analysis to assess the efficacy of Buyang Huanwu Decoction combined with acupuncture for the treatment of stroke sequelae.

## 2 Methods

The current protocol is prepared in accordance with the Preferred Reporting Items for Systematic reviews and Meta-Analyses for Protocols statement guideline. This protocol has been registered on Open Science Framework, with the unique registration number 10.17605/OSF.IO/W8BF5.

### 2.1 Search strategy

Seven electronic databases including Web of Science, Embase, PubMed, Wanfang Data, Scopus, Science Direct, and Cochrane Library were searched in August 2021 by 2 independent reviewers. The following search terms are used: “Buyang Huanwu Decoction”, “acupuncture”, “stroke of sequelae”, “stroke of apoplexy”, and “random”. The reference lists of the included studies were also checked for additional studies that were not identified with the database search. There was no restriction in the dates of publication or language in the search. No ethical approval was required in our study because all analyses were based on aggregate data from previously published studies

### 2.2 Eligibility criteria

If these studies meet the following criteria, they are analyzed and studied: the included studies are randomized controlled trials; the participants in the treatment group were treated with Buyang Huanwu Decoction combined with acupuncture and the control group were treated with acupuncture alone; diagnosis after Computed Tomography or Magnetic Resonance Imaging; the main therapeutic indicators: clinical efficacy rate and adverse reaction rate. Exclusion criteria include: the treatment group adopts simple Western medicine treatment or single Chinese medicine treatment; the details of the test are unclear, or the result data are incomplete; the same test data are repeatedly published or animal tests; the test data are obviously defective or incompletely provided, and the test protocol design is not reasonable; there were less than 50 patients in each literature.

### 2.3 Data extraction

Two independent authors will extract the following descriptive raw information from the selected studies: study characteristics such as author, study design, study language, publication year, mean follow-up period; patient demographic details such as number, average age, body mass index, and gender ratio; details of interventions, and outcome measures. If the data are missing or cannot be extracted directly, we will contact the corresponding authors to ensure that the information is integrated. Otherwise, we will calculate them with the guideline of Cochrane Handbook for Systematic Reviews of Interventions 5.1.0. If necessary, we will abandon the extraction of incomplete data.

### 2.4 Quality assessment

The risk of bias assessment of the included studies was performed by 2 authors independently using the tool recommended in the Cochrane Handbook for Systematic Reviews of Interventions (version 5.1.0).^[11]^ This tool included 7 aspects which were sequence generation (selection bias), allocation sequence concealment (selection bias), blinding of participants and personnel (performance bias), blinding of outcome assessment (detection bias), incomplete outcome data (attrition bias), selective outcome reporting (reporting bias), and other bias (baseline balance and fund). Additionally, each of the aspects was ranked low risk of bias, high risk of bias, and unclear risk of bias.

### 2.5 Statistical analysis

We will perform meta-analysis using STATA 11.0 (http://www.stata.com; Stata Corporation, College Station, TX). The Q-test and I^2^ values will be used to indicate inter-study heterogeneity. When the *P* value of Q-test >0.1 and I^2^ <50%, a fixed-effects model was applied; otherwise, a random-effects model was used. Binary variables were expressed by odds ratio with 95% confidence interval, and continuous variables by mean difference with 95% confidence interval. If significant heterogeneity is found, we will try to explore the source of heterogeneity by subgroup analysis based on specified effect modifiers as follows: interventions, publication year, participant's average age, sample size, publication language, and so on.

## 3 Discussion

Stroke is a substantial health threat and one of the leading causes of disability worldwide, representing a huge burden to the patient's family and to society.^[12,13]^ With the acceleration of social aging, the incidence of stroke increases year by year, prompt and effective treatment is of great significance for reducing the mortality and sequelae.^[14,15]^ The benefits of acupuncture and Buyang Huanwu Decoction have been proven for many diseases, including stroke sequelae. This is the first protocol to evaluate the efficacy of Buyang Huanwu Decoction combined with acupuncture in the treatment of patients with stroke sequelae. The findings of this study will provide scientific evidence for clinician and future research who work in this field of knowledge.

In future research, we will focus on 3 main issues that should be considered in traditional Chinese medicine study: the sample size should need to be based on sufficient statistical data, and the calculation method of the sample size method should be reported in the text; the random method needs to be fully described and appropriately hidden; some drugs with obvious therapeutic effects and some ineffective or harmful conventional drugs should be recorded in detail; future clinical trials should pay attention to the occurrence of more adverse events, especially for long-term treatment safety studies. Adverse events should adopt international standards medical terms are recorded and reported.

## Author contributions

Liang Zhang plans the study design. Zhaowei Li reviews the protocol and collects data. Jiayu Zhang writes the manuscript. All authors approve the submission.

**Conceptualization:** Zhaowei Li.

**Funding acquisition:** Liang Zhang.

**Writing – original draft:** Jiayu Zhang.
